# Ultrasonic neuromodulation mediated by mechanosensitive ion channels: current and future

**DOI:** 10.3389/fnins.2023.1232308

**Published:** 2023-07-31

**Authors:** Mengyao Song, Mingxia Zhang, Sixuan He, Le Li, Huijing Hu

**Affiliations:** ^1^Institute of Medical Research, Northwestern Polytechnical University, Xi’an, China; ^2^Research and Development Institute of Northwestern Polytechnical University in Shenzhen, Shenzhen, China

**Keywords:** ultrasound, neuromodulation, Piezo ion channels, transient receptor potential channels, mechanosensitive ion channels

## Abstract

Ultrasound neuromodulation technology is a promising neuromodulation approach, with the advantages of noninvasiveness, high-resolution, deep penetration and good targeting, which aid in circumventing the side effects of drugs and invasive therapeutic interventions. Ultrasound can cause mechanical effects, activate mechanosensitive ion channels and alter neuronal excitability, producing biological effects. The structural determination of mechanosensitive ion channels will greatly contribute to our understanding of the molecular mechanisms underlying mechanosensory transduction. However, the underlying biological mechanism of ultrasonic neuromodulation remains poorly understood. Hence, this review aims to provide an outline of the properties of ultrasound, the structures of specific mechanosensitive ion channels, and their role in ultrasound neuromodulation.

## Introduction

Ultrasound refers to sound waves with frequencies above 20,000 Hertz (Hz), which exceeds the human hearing range of 20 Hz–20 kHz. With its high frequency and short wavelength, ultrasound has linear propagation capabilities within a finite distance, high beam focusing and excellent directionality features (Dell'Italia et al., 2022). Moreover, ultrasound is a noninvasive technique for neural regulation. It has high safety and can be employed in combination with magnetic resonance imaging. Exosomes produced by human astrocyte (HA) cells stimulated by ultrasound were comparable in terms of size distribution and morphology with those of untreated HA cells (Deng et al., 2021). Moreover, HE staining conducted after 4 weeks of ultrasound treatment showed no major organ damage, which further affirmed the safety of ultrasound therapy in human. Neuroregulatory technology, such as transcranial ultrasound stimulation, transcranial magnetic stimulation, transcranial electrical stimulation, deep brain stimulation, and optogenetics, is a kind of therapeutic approach that implements either implantable or nonimplantable devices to alleviate patients’ symptoms. A comparison of these different neural regulation modes is shown in Table 1 below. It can effectively modulate the activity of the central, peripheral or autonomic nervous systems by either physical (e.g., light, sound, electricity, magnetism, etc.) or chemical (e.g., drugs) means (Jiang et al., 2019). Currently, ultrasound is frequently involved in treatment procedures as a physical factor. High-intensity ultrasound is typically recommended for tumor ablation, additionally, it has been applied to treat several neurological diseases including Parkinson’s disease, essential tremor and obsessive–compulsive disorder (Sperling et al., 2018; Germann et al., 2021; Iorio-Morin et al., 2021). Low-intensity ultrasound is often used for neural regulation.

**Table 1:** Comparison of several neural regulation modes.

	Transcranial ultrasound stimulation (TUS)	Transcranial magnetic stimulation (TMS)	Transcranial electrical stimulation (TES)	Deep brain stimulation (DBS)	Optogenetics
Whether it is invasive or not	Noninvasive	Noninvasive	Noninvasive	Invasive	Invasive
Depth of action	Deep	Shallow, difficult to stimulate subcortical tissue	Shallow, difficult to stimulate subcortical tissue	Deep	The maximum stimulation depth of the two-photon optogenetic system is 400 μm.
Spatial resolution, precision	mm	>1 cm	cm	μm	Subcellular precision
The target/mode of action	Cortex (primary motor cortex (M1), somatosensory, primary visual cortex), deep brain (e.g., hippocampus, thalamus)	M1 area, SMA area, bilateral dorsolateral prefrontal cortex (DLPFC)	M1 area, DLPFC	Stereotactic implanted electrodes provide chronic electrical stimulation to specific targets in the brain	Specific neurons expressing the photosensitive protein can be induced via viral vector or transgenic means. When illuminated by different wavelengths and frequencies of light, these neurons become depolarized or hyperpolarized, leading to neuron excitation or inhibition.
Security	High and can be used in conjunction with magnetic resonance	rTMS has high safety, high frequency rTMS treatment may induce epilepsy (low probability).	Weak current do not induce action potential, will not induce seizures, high safety.	Batteries need to be replaced regularly, and there are surgical complications such as bleeding and infection.	Optical fiber implantation will still inevitably cause brain tissue damage and local bleeding to a certain extent, especially when large-diameter optical fibers are used for high-intensity light input.

The effects of ultrasound mainly result from mechanical, cavitation and thermal effects (Darmani et al., 2022). Mechanical effects stem from ultrasound functioning as a mechanical wave, exerting radiation forces on biological tissues. Acoustic radiation force (ARF) can cause mechanical vibration and cell membrane deformation, and activate mechanosensitive ion channels in neurons, and discharge cells (Peng et al., 2020). On the other hand, thermal effects refer to biological tissue cells absorbing the energy of ultrasound, causing a subsequent temperature increase and mediating cell excitability. However, too high a temperature denatures enzymes and proteins, leading to decreased biological activity. Moreover, cavitation effects arise from the formation of small gas bubbles in tissue under positive and negative pressure phases of ultrasound oscillation stretching (Baek et al., 2017; Blackmore et al., 2019; Feng et al., 2019). Currently, it is commonly surmised that the biological impacts of ultrasound are primarily attributed to its mechanical influence rather than its thermal attributes.

Mechanosensitive (MS) ion channels refer to a group of transmembrane channel proteins that can convert mechanical stimuli signals into electrical or chemical signals. Mechanically-sensitive channels exist widely in bacterial, archaea, and eukaryotic organisms (Martinac, 2004). In bacteria and archaea, mechanosensitive channels serve as protection and survival mechanisms. Their primary function is to release intracellular substances as an emergency valve to lower osmotic pressure when the extracellular environment becomes hypotonic such as during heavy rain (Ajouz et al., 1998). The discovery of new families of mechanically activated ion channels, such as PIEZO, which have important *in vivo* physiological roles in mammals, opens new avenues for studying the role of mechanotransduction in human health and disease (Coste et al., 2010; Murthy et al., 2017; Kefauver et al., 2020). In 2016, Kubanek reported that ultrasound triggered a current via the heterologous expression of two pore domain potassium channels (K2P channels) including TREK-1, TREK-2, TRAAK and voltage-gated sodium channels (Nav1.5) in the *Xenopus* oocyte system (Kubanek et al., 2016). Heterologous expression of TRP-4 channels in *Caenorhabditis elegans* neurons revealed that their motion behavior was provoked by ultrasound. These discoveries suggest that the mechanical force generated by ultrasound could open mechanosensitive ion channels in the cell membrane and induce ion flow, which can alter neuronal excitability and eventually result in biological effects.

Despite substantial recent progress in the identification and characterization of mechanically activated ion channels, a variety of biological processes that depend on mechanotransduction remain poorly understood at the molecular level, and the identities of many mechanosensors remain elusive. Here, this article will first review the structures of several mechanosensitive ion channels and then outline the progress in their research related to ultrasound to help bolster our understanding of mechanotransduction at the molecular level.

### Mechanosensitive ion channels of large conductance

#### Structure of mechanosensitive ion channels of large conductance

Sukharev et al. (1994) discovered two types of mechanosensitive ion channels in *E. coli*, which were named based on their pore size: the mechanosensitive channel of large conductance (MscL) and the mechanosensitive channel of small conductance (MscS). Perozo et al. (2002) demonstrated that the open state of MscL is highly dynamic, supporting a water-filled pore of at least 25 Å, lined mostly by the first transmembrane helix (TM1), allowing passage of large organic ions and small proteins. The patch-clamp electrical recording technique showed that when the patches were subjected to suction or solution bathing the patch was diluted, and voltage-controlled (−clamped) patches of *E. coli* membranes produced giant steps in unitary current (Martinac et al., 1987; Sukharev et al., 1993; Levina et al., 1999). MscS showed a single channel conductance of ~1 nS and demonstrated both pressure and voltage dependence, and selectivity for anions. While MscL with ~3 nS conductance when subjected to even stronger suction.

MscL is widely expressed in prokaryotic cells but is not present in eukaryotes. MscL has been extensively studied by scientists in recent years. Chang and colleagues indicated that the gating of MscL is primarily regulated by lipoprotein interactions (Chang et al., 1998). The composition of MscL comprises five identical subunits forming an ion channel, which is in turn regulated by membrane tension. The three-dimensional structure of the *Mycobacterium tuberculosis* MscL homolog was determined through X-ray crystallography, to a resolution of 3.5 angstroms. Each subunit consists of 136 amino acid residues, alongside two transmembrane α-helical regions (TM1 and TM2), and a loop region that links them on the extracellular side. The termini (N-terminal and C-terminal) are found on the cytoplasmic surface. The region responsible for pore formation by MscL results from TM1 of the five constituent subunits, and TM2 primarily associates with the cytoplasmic membrane lipid bilayer. MscL is a mechanically gated ion channel, featuring considerable conductance and no ion selectivity; physiologically, the protein structure resembles a cylinder. Additionally, MscL can be expressed stably in the cell membranes of eukaryotic cells.

#### Research on mechanosensitive ion channels of large conductance in ultrasonic neural modulation

The MscL channel is expressed in prokaryotes and its function in these cells has been extensively researched. However, the application and study of MscL in eukaryotes is just emerging. MscL channels can be expressed in eukaryotic cell lines including CHO and HEK293 cells (Doerner et al., 2012). Through electrophysiological experiments and dye release experiments, the authors demonstrated the mechanosensitive function of the MscL channels. Ye et al. (2018) also confirmed the channel activity of MscL in HEK293T cells and neurons through electrophysiological experiments. However, MscL channels expressed in eukaryotic systems have a lower opening threshold and smaller conductance than those in prokaryotes. Therefore, the authors expressed the functional mutant I92L MscL in neurons. Fluorescent localization and electrophysiological experiments proved that MscL I92L can be expressed and inserted into the neuronal membrane and has channel activity. Furthermore, MscL I92L demonstrated greater sensitivity to mechanical stimulation than the wild-type MscL channel. This is exemplified by only 30 mmHg of force leading to significant channel opening.

### Piezo ion channels

#### Structure of Piezo ion channels

Piezo ion channels refer to a group of channels discovered and named by Coste et al. (2010). Piezo1 (Fam38A) was identified as the ion channel essential for generating mechanosensitive potentials in the Neuro2A cell line through expression profile and RNA interference knockdown of candidate genes techniques. Only two types of Piezo families have been discovered so far, Piezo1 (Fam38A) and Piezo2 (Fam38B). Piezo1 is a nonselective cationic channel that can be inhibited by GsMTx4 (tarantula venom), gadolinium (Gd), and ruthenium red (RR) (Copp et al., 2016). Piezo2, a mammalian cognate of Piezo1, records mechanically sensitive electrical currents in separated dorsal root ganglion (DRG)neurons. Piezo1 channels mainly exist in nonsensory tissues, including the skin, lungs, kidneys, and bladder. On the other hand, Piezo2 channels occur mainly in sensory tissues such as the trigeminal ganglion (TG), DRG sensory neurons, and Merkel cells.

Each subunit of Piezo proteins is made up of over 2000 amino acid residues, and their molecules are relatively large, having a molecular mass of 1.2 × 10^^6^. Ge et al. determined that the full-length of the cryo-electron microscopy structure is 2,547 amino acids, with a resolution of 4.8 Å in mouse Piezo1 (Ge et al., 2015). The findings reveal that Piezo1 consists of a triple helix structure, with extracellular domains comprising three distal blades and a central cap. There are 14 apparently resolved segments per subunit in the transmembrane region that form three peripheral wings and a central pore module that encloses a potential ion-conducting pore. The carboxyl terminal is responsible for the pore section of the ion channel, while the amino terminal receives mechanical stimulation that opens the carboxyl terminal pore.

#### Research on Piezo ion channels in ultrasonic neural regulation

Piezo1 activation plays an essential role in mechanical transmission via ultrasonic stimulation. Qiu et al. (2019) conducted a study that revealed that the activation of heterologously expressed HEK293T cells and endogenous Piezo1 channels can be achieved through low-intensity and low-frequency ultrasound stimulation. This resulted in Ca^2+^ influx as well as increased nuclear c-Fos expression levels in primary neurons, although when pre-treated with a Piezo1 inhibitor the effect was inhibited in cells. Furthermore, the study demonstrated that ultrasonic stimulation significantly affected downstream Ca^2+^ signaling protein levels and induced the expression of important proteins such as phospho-CaMKII, phospho-CREB, and c-Fos in a neuronal cell line. These proteins are known to play significant roles in complex neuronal functions like learning, memory, and neuronal plasticity. Notably, the impact of ultrasonic stimulation on Ca^2+^ signaling protein levels was found to decrease with the loss of Piezo1 channel functions. In 2023, the authors also reported that Piezo1 knockout (P1KO) in the right motor cortex of mice significantly decreased ultrasound-induced neuronal calcium responses, limb movement, muscle electromyogram (EMG) signaling and C-Fos expression compared to the control. Central amygdala (CEA) neurons, having higher Piezo1 expression levels, displayed greater sensitivity to ultrasound than cortical neurons. Piezo1 is expressed in both neurons and astrocytes. The authors demonstrated that Piezo1 is expressed in different brain regions and that neuronal Piezo1 plays an important role in mediating ultrasound effects directly (Shen et al., 2021). The researchers also developed a Piezo1-targeted microbubble (PTMB) which can bind to the extracellular domains of the Piezo1 channel (Zhu et al., 2023).

### Transient receptor potential channels

#### Structure of transient receptor potential

The discovery of the transient receptor potential (TRP) channel initially occurred within the visual system of *Drosophila melanogaster*, and was based upon the peculiar behavior of mutant Drosophila in response to sustained light exposure. This resulted in the production of transient potentials as opposed to sustained peak potentials. TRP channels have been found to be highly conserved genes across a broad spectrum of species, ranging from *Caenorhabditis elegans* to humans. They exhibit prominent distribution within sensory neurons and play crucial roles in the modulation of external mechanical stimuli, including pressure and sound waves. Additionally, these channels are implicated in the generation of senses associated with touch, pain, hearing, taste, and vision (Voolstra et al., 2010).

TRP channels are nonselective cationic channels that are highly permeable to Ca^2+^ and Na^+^, with TRPM6 and TRPM7 being highly permeable to Mg^2+^. Nilius and Owsianik (2011) classified TRP ion channels into seven subtypes and two categories based on amino acid sequences and three-dimensional structures. The first category includes TRPC (TRP-canonical), TRPV (TRP-vanilloid), TRPM (TRP-melastatin), TRPA (TRP-ankyrin) and TRPN (TRPNompC), whereas the second category includes TRPP (TRP-polycystin) and TRPML (TRP-mucolipin). The TRP channel family consists of nonselective cationic channels that are made up of four tetramer monomers. These monomers contain a hexaxial transmembrane (TM) domain with a pore ring structure located between TM5 and TM6.

#### Research on transient receptor potential channels in ultrasonic neural regulation

TRP-4 channels are primarily expressed in four CEPs including CEPDL, CEPDR, CEPVL and CEPVR dopaminergic neurons and in two ADEs consisting of ADEL and ADER dopaminergic neurons, and DVA and DVC interneurons in a few *C. elegans* neurons. Ibsen et al. (2015) conducted a study in which they observed the effect of defective TRP-4 mutation in nematodes with respect to their response to ultrasound-combined microbubbles. The mutant nematodes exhibited a reduction in large reversal responses compared to the wild type. Subsequently, researchers transferred the TRP-4 gene to amphid wing ‘C’ (AWC) neurons leading to its induction. This activation of TRP-4 gene expression in AWC neurons was found to result in the accumulation of calcium ions under specific peak negative pressures (0.41 and 0.47 MPa) of ultrasonic stimulation. This response was not observed in the wild-type AWC cells. Additionally, the decrease in contrarian motor behavior in *C. elegans* mutants with TRP-4 indicated that the induction of TRP-4 by ultrasonic stimulation (peak negative pressure < 0.5 MPa) might regulate the activity of neurons involved in reverse motor behavior, thereby leading to a reduction in reverse movement.

### Two-pore-domain potassium channels

#### Structure of two-pore-domain potassium channels

The identification of the K2P channel first occurred in the human kidney, as its characteristic two pore regions prompted its labeling as a two-pore-domain potassium (K2P) channel. This particular form of channel is capable of activation along the entire range of physiological voltages, elucidating both the background potassium current and the baseline potassium current, and is not vulnerable to typical potassium channel blockers (Lesage et al., 1996).

Potassium channels are a diverse group of proteins that play a crucial role in regulating cellular activity and maintaining cellular homeostasis. These channels can be classified into three main types based on their structure and function: calcium-activated (KCa) potassium channels, two-pore-domain potassium channels (K2P) and inward-rectified potassium channels (Kir). Among these, K2P channels have gained particular attention in recent years due to their unique structure and function. Currently, K2P channels are categorized into six subgroups or “clades” each with distinct structural and functional characteristics. These six clades are: TWIK, TASK, TREK, TALK, THIK, and TRESK. Notably, only the TREK subgroup of K2P channels is known to be mechanically sensitive. Thus, understanding the distinct properties of each K2P channel subtype is critical for gaining insight into their role in physiological and pathological processes (Feliciangeli et al., 2015). Kv, KCa, and Kir are tetrameric channels, with each monomer having a single pore domain. However, K2P is a dimeric channel, and each monomer is composed of two pore regions. These channels consist of two pore domains (P1 and P2), two extracellular cap helices (C1 and C2), and four transmembrane domains (M1–M4) with both the amino and carboxyl terminus situated on the cytoplasmic side (Renigunta et al., 2015). K2P channels which are equipped with the capacity to perceive mechanical stimulation at the cellular membrane are classified as mechanically sensitive channels, namely TREK-1, TREK-2, and TRAAK. The high-resolution crystal structures of TWIK-1, TRAAK, and TREK-2 channels have been made available. These structures divulge the existence of multiple helices within the extracellular ring between TM1 and the P1, resulting in the formation of a physical obstruction that urges ions to exit via the side pore.

#### Research on two-pore-domain potassium channels in ultrasonic neuromodulation

Sorum et al. (2021) proposed that ultrasound has the potential to trigger the opening of mechanosensitive TRAAK channels through an increase in membrane tension. This study provides insight into the vital role of mechanosensitive channels in physiological responses to ultrasound and presents a promising avenue for gene targeting in the regulation of cellular auditory nerves. The authors observed that the application of short-pulsed, low-intensity ultrasound (10 ms, 5 MHz, 1.2 W/cm^2^) led to a swift and robust activation of TRAAK channels in plaques of *Xenopus oocytes* expressing TRAAK as well as in cortical neurons of mice also expressing TRAAK. The K^+^ selective ultrasonic stimulation current featured a reversal potential that was proximal to the Nernst equilibrium potential for K+ (EK^+^ = −59 mV), comparable to the TRAAK currents for both base and pressure stimulation. In contrast, the study revealed that the non-mechanosensitive K2P ion channel TASK2 was not activated by ultrasound.

In addition to its role in neuromodulation, ultrasound also has various applications, such as reducing fracture healing time (especially in delayed healing and bone nonunion), preventing inflammatory loosening of prosthetics, and promoting tendon, ligament, and cartilage recovery. Furthermore, it can inhibit lipopolysaccharide-induced inflammation and reduce proinflammatory factors (Chan et al., 2010; Jeremias Júnior et al., 2011; Loyola-Sánchez et al., 2012; Ren et al., 2013; Zhao X et al., 2017; Jiang et al., 2019). Table 2 summarizes the research progress on the above mechanically sensitive ion channels related to ultrasonic neural regulation.

**Table 2:** Studies on several mechanosensitive ion channels in ultrasonic neuromodulation.

Types of mechanosensitive ion channels	Author	Subject or site of ultrasonic stimulation	Main results	References
MscL	Ye et al.	Primary cultured rat hippocampal neurons and functional gain mutation I92L and HEK293T cells	MscL was expressed in primary cultured rat hippocampal neurons and demonstrated to be activated by low pressure ultrasonic pulses. I92L countersensitizes the MscL to ultrasound, triggering action potentials at a peak negative pressure as low as 0.25 MPa.	Ye et al. (2018)
	Qiu et al.	Neurons in the cerebral cortex or dorsomedial striatum of mice	Ultrasound triggered Ca^2+^ influx in 293T cells expressing MscL-G22S and activation of downstream neurons. Non-invasive ultrasound triggered neural activation in MscL-G22S expression regions, and c-Fos was significantly upregulated without widespread nonspecific activation. Rapid electromyographic response induced by ultrasound targeting MscL in M1 region of cortex; Ultrasound successfully activated MscL-expressing neurons in the deep DMS region.	Qiu et al. (2020)
	Heureaux et al.	Wild-type MscL and G22S mutant activated, retinal pigment epithelial cells (RPE)	Ultrasonic-driven integrin-bound microbubbles can cause MscL opening. The activation of MscL induced by acoustic tweezing cytometry (ATC) depends on the functional connection of microbubbles with the intact actin cytoskeleton.	Heureaux et al. (2014)
Piezo	Pan et al.	HEK293T cells, Jurkat T-cells and primary T cell (peripheral Blood mononuclear Cells, PBMCs)	Successful expression of the ion channel Piezo1 in HEK293T cells and subsequent Ca^2+^ influx triggered the downstream pathway for gene expression. The Piezo1 gene was transferred into Jurkat T cell lines and PBMCs to create chimeric antigen receptors, transactivated to open channels and stimulate ultrasound response. ReCoM is effective in controlling CAR expression in T cells to guide the recognition and eradication of tumor cells for controllable cancer immunotherapy.	Pan et al. (2018)
	Qiu et al.	HEK293T cells, mouse primary cortical neurons, mouse hippocampal cell line mHippoE-18 (CLU199), HeLa cells	The ultrasound alone activated both heterologous and endogenous Piezo1, initiating calcium influx and increased the expression of nuclear c-Fos in primary neurons, but not when pre-treated with Piezo1 inhibitor GsMTx-4. Ultrasound significantly augmented the expression of critical proteins including phospho-CaMKII, phospho-CREB, and c-Fos in neuronal cell lines. However, the downregulation of Piezo1 notably decreased this effect.	Qiu et al. (2019)
	Shen et al.	Neuro2A cell lines, rat hippocampal neurons	US energy can reach comparable levels of cytoplasmic Ca^2+^ transients at a peak negative pressure of 0.03 MPa in Piezo1-targeted microbubble (PTMB)-binding cells, whereas control cells typically require US intensity of 0.17 MPa. The cytoplasmic Ca^2+^ elevation was greatly reduced by chelating extracellular calcium ions or by using cationic ion channel inhibitors such as GsMTx-4, confirming that US-mediated calcium influx are dependent on the Piezo1 channels. Cavitation and heating effects of US hardly participate in the process of Ca^2+^ transients.	Shen et al. (2021)
	Zhu et al.	Conditional knockout mouse model, central amygdala (CEA) neurons, cortical neurons	Piezo1 knockout (P1KO) in the right motor cortex of mice significantly decreased ultrasound-induced neuronal calcium responses, limb movement, muscle electromyogram (EMG) signalings and C-Fos expression compared to the control. CEA neurons, having higher Piezo1 expression levels, displayed greater sensitivity to ultrasound than cortical neurons. Piezo1 expressed in both neurons and astrocytes. They demonstrated that Piezo1 expressed in different brain regions and neuronal Piezo1 played an important role in mediating ultrasound’s effects directly.	Zhu et al. (2023)
	Zhang et al.	MC3T3-E1 cells	Piezo1 can transmit LIPUS-induced mechanical signals to intracellular calcium. Ca^2 +^ influx acts as a second messenger to activate ERK1/2 phosphorylation and perinuclear F-actin filament polymerization, regulating MC3T3-E1 cells proliferation.	Zhang et al. (2021)
TRP	Ibsen et al.	*Caenorhabditis elegans*	Low-pressure ultrasound (with peak negative pressures of 0.4–0.6 MPa) specifically activated neurons expressing the TRP-4 channel. Misexpressing TRP-4 in ASH and AWC sensory neurons resulted in an increase in large reversals, while misexpressing it in PVD neurons suppressed this behavior, a novel role for this neuron.	Ibsen et al. (2015)
	Magaram et al.	*Caenorhabditis elegans*	Ultrasonic stimulation induced a reversal response in *C. elegans* by the pore-forming TRP-4 subunit and the DEG/ENaC/ASIC ion channel MEC-4. Under lower pressure (0.79 MPa), the expression of TRP-4 in AWC chemosensory neurons partially rescued the reversal of TRP-4 (ok1605) and Mec-4 (u253) mutants, in contrast to ASH, which only reversed at a higher pressure (>0.92 MPa).	Magaram et al. (2022)
	Oh et al.	Astrocyte, HEK293T, and HEK293T-piezo1 knockout (HEK-P1KO) cells	Low intensity low-frequency ultrasound (LILFU) induced neuromodulation by opening TRPA1 channels in astrocytes. The influx of Ca^2+^ caused a release of glial transmitters, including glutamate via Best1 channels. The released glutamate activated NMDA receptors in neighboring neurons and triggered action potential generation.	Oh et al. (2019)
	Duque et al.	C57BL/6J mice, Npr3-cre mice, Npr3-cre mice, Bl/6 male mice, Balb/c mice, TRPA1 knockout mice	Human transient receptor potential A1 (hs TRPA1) is ultrasonically sensitive to mammalian HEK cells and rodent neurons *in vitro* and *in vivo*. Ultrasound evoked gating of hsTRPA1 specifically requires its N-terminal tip region and cholesterol interactions. hs TRPA1 enhanced ultrasound-induced calcium transients and activated ultrasound-induced action potentials in primary neurons in rodents. Unilateral expression of hsTRPA1 in mouse layer V motor cortical neurons leads to c-fos expression and contralateral limb responses in response to ultrasound delivered through an intact skull.	Duque et al. (2022)
	Yoo et al.	Primary cortical neurons	Ultrasound stimulation triggers calcium entry across the plasma membrane. TRPP1/2, TRPC1, and Piezo1 as mechanosensitive ion channels involved the ultrasound response. Overexpression of TRPC1, TRPP2, and TRPM4 increased the sensitivity of cortical neurons to ultrasound with reduced pulse intensities and durations, in the case of TRPM4 greatly accelerated the response kinetics.	Yoo et al. (2022)
	Burks et al.	Female C3H, SV129 or TRPC1tm1Lbi/Mmjax (TRPC1 knockout) mice, C2C12 muscle cells or TCMK1 kidney cells	Inhibiting VGCC or TRPC1 *in vivo* prevented COX2 upregulation and the migration of MSCs to kidneys and muscle in response to PfUS. A TRPC1/VGCC complex was observed in plasma membranes. The inhibition of VGCC or TRPC1 blocked pFUS-induced Ca^2+^ transients in TCMK1 and C2C12 cells. The mechanical activation of the Na^+^ TRPC1 current upstream of VGCC was found to be caused by the pFUS acoustic radiation force, instead of direct opening VGCC.	Burks et al. (2019)
K2P	Zhao et al.	PC12 cells	Pre-treatment with LIPUS (1 MHz, 50 mW/cm^2^, 20% duty cycle and 100-Hz pulse repetition frequency, 10 min) inhibited MPP1-induced neurotoxicity and mitochondrial dysfunction in PC12 cells. LIPUS regulated the expression of antioxidant proteins, specifically thioredoxin-1 and heme oxygenase-1, decreasing oxidative stress induced by MPP+. The prevention of neurocytotoxicity was observed through the activation of pathways that involved the phosphoinositide 3-kinase (PI3K)-Akt and extracellular signal-regulated kinase (ERK1/2). LIPUS protected neuronal cells from MPP + -induced cell death through the K2P channel- and stretch-activated ion channel-mediated downstream pathways.	Zhao L. et al. (2017)
	Kubanek et al.	*Xenopus* oocytes	Focused ultrasound (10 MHz, 0.3–4.9 W/cm^2^) modulated the currents flowing through the ion channels averaged up to 23%, depending on channels and stimulus intensity. Repeated stimulation of the channel led to a reversible effect that decreased when the K2P channel was subjected to the blocking effect of BaCl_2_. At the single cell level that focused US modulates the activity of specific ion channels to mediate transmembrane currents.	Kubanek et al. (2016)
	Sorum et al.	*Xenopus laevis* Oocytes, *Pichia pastoris* cells	Ultrasonic energy is transduced to TRAAK through the membrane in a manner analogous to canonical mechanical activation, likely increasing membrane tension to promote channel opening. Ultrasounic had an effect on modulation of neuronal expression TRAAK. These results suggest that mechanosensitive channels play a ctitical role in physiological responses to ultrasound and can be used as tools for acoustic neuromodulation of genetically targeted cells.	Sorum et al. (2021)
	Prieto et al.	CA1 pyramidal neurons in acute rodent hippocampal brain	Focused high-frequency (43 MHz) ultrasound inhibits or enhances firing in a spike frequency-dependent manner. Ultrasound increases the threshold current of action potential firing, the slope of the frequency input curve, and the maximum firing frequency. Ultrasound mildly hyperpolarizes the resting membrane potential, reduces action potential width, and increases the depth of after-hyperpolarization. Ultrasound activates thermosensitive and mechanosensitive two-pore-domain potassium (K2P) channels through acoustic radiation force-induced heating or mechanical effects. Finite element modeling shows that ultrasound affects the firing frequency of brain tissue by slightly raising the temperature (<2°C) and possibly through mechanical effects.	Prieto et al. (2020)

## Summary

Mechanosensitive ion channels are mechanical force molecular sensors that are activated by mechanical stimuli and located on the cell membrane. These channels can quickly and efficiently convert mechanical stimuli into electrical and chemical signals. Stretching of mechanosensitive proteins can damage molecular binding sites, expose regulatory sites, and change the association or dissociation rates for protein binding (Ingham et al., 1997; Krammer et al., 2002; Cain et al., 2021). MS channel opening can result in ion influx. Additionally, numerous diseases such as muscular dystrophy, and cardiac arrhythmias and et al. have been related to defects in activating MS ion channels (Sukharev and Sachs, 2012). However, the gating mechanism and physiological effects of these channels differ. We also do not know the connection between the structure and the function of these MS ion channels. Ultrasound usually activates more than one MS ion channel, but it is unknown which one is working or how they work together. Our understanding of the mechanisms and functions of ultrasound activated mechanosensitive ion channels remains limited.

We need to develop innovative research methods and conduct more thorough research on the mechanism of these channels and explore screening of mechanically sensitive ion channels or their mutants that can be accurately controlled in the future. Addtionally, these MS ion channels should be expressed in various nerve cells. In future studies, researchers should clarify the expression profile of each ion channel in neuronal cells, and then study their sensitivity to ultrasound modulation, so as to summarize and compare the differences in the role of various ion channels in ultrasound modulation.

To date, previous studies have demonstrated the structure of some specific MS ion channels and their investigated their roles in ultrasonic neuromodulation. Ultrasound offers several advantages, including non-invasiveness, convenient *in vitro* regulation, intracranial multipoint focusing, spatiotemporal controllability and accuracy. Basic clinical trials have demonstrated that ultrasound can improve specific behaviors, such as increased responsiveness in patients with chronic disorders of consciousness (Cain et al., 2021) and improved mood (Reznik et al., 2020; Sanguinetti et al., 2020). However, further studies are needed to investigate how ultrasound acts on organisms, which mechanically sensitive channels it influences, and how different ultrasonic stimulation parameters produce varying effects. To gain a better understanding of ultrasonic neuromodulation efficacy, it is necessary to conduct these studies in various species and different disease models, and set different ultrasonic stimulation parameters to observe the applicability, persistence and timeliness of ultrasonic stimulation. It is also crucial to address the thermal and cavitation effects of ultrasonic stimulation and improve ultrasonic focusing resolution to increase the precision of ultrasonic regulation. Finally, innovative ultrasonic equipment should be developed to enhance the efficacy and applicability of wearable ultrasonic equipment.

## Author contributions

HH: conceptualization. MS and MZ: writing—original draft preparation. MS, MZ, SH, LL, and HH: writing—review and editing. All authors contributed to the article and approved the submitted version.

## Funding

This work was supported by the Natural Science Foundation of Shaanxi province (2022-JM482), Shenzhen Science and Technology Program (GJHZ20210705143401005), the Education and Teaching Reform Funds for the Central Universities (No. 23GZ230102), and the National Natural Science Foundation of China (No. 31771016).

## Conflict of interest

The authors declare that the research was conducted in the absence of any commercial or financial relationships that could be construed as a potential conflict of interest.

## Publisher’s note

All claims expressed in this article are solely those of the authors and do not necessarily represent those of their affiliated organizations, or those of the publisher, the editors and the reviewers. Any product that may be evaluated in this article, or claim that may be made by its manufacturer, is not guaranteed or endorsed by the publisher.
